# Trends in Dementia Prevalence in the United States, 1995-2019: An Age-Period-Cohort-Interaction Analysis

**DOI:** 10.1093/geroni/igab046.287

**Published:** 2021-12-17

**Authors:** Mark Lee, Liying Luo

**Affiliations:** 1 University of Minnesota, Minneapolis, Minnesota, United States; 2 Pennsylvania State University, University Park, Pennsylvania, United States

## Abstract

Previous studies have indicated that age-specific dementia prevalence has declined in the United States and other high-income countries. However, these studies have been limited by estimating temporal change in dementia rates on a strictly period basis, with little attention to possible cohort effects. Distinguishing age, period, and cohort effects is both methodologically and theoretically important for identifying the etiological factors driving dementia decline in the population. In this study, we apply the novel Age-Period-Cohort-Interaction (APC-I) model, which defines cohort effects as the interaction between age and period main effects. The APC-I model improves on earlier APC models (e.g., the accounting method) by solving the linear dependence between predictors through theoretical clarification instead of statistical manipulation. We use the APC-I model to estimate period and cohort trends in dementia prevalence using data from the Health and Retirement Study (HRS). Our analysis points to significant period and cohort effects. Dementia prevalence declined significantly between the periods 1995-1999 and 2015-2019. At the same time, cohorts born in the 1940s had significantly lower odds of dementia than would be expected given age and period main effects. This cohort’s unique protection from dementia has been relatively stable as they have aged. Our study identifies the periods during which and cohorts for whom dementia risk has declined in the United States. Further research is needed to specify the period factors (e.g., broad based improvements in nutrition) and cohort factors (e.g., increases in educational attainment) that are responsible for these trends.

